# Responses of coal tits (*Periparus ater*) to aversive food: insights into hoarding motivation and memory

**DOI:** 10.1007/s10071-025-01969-8

**Published:** 2025-06-05

**Authors:** D. D. O’Hagan, D. Donley, S. W. Y. Yeung, C. D. Blasi Foglietti, D. Wales, D. Wintersgill, T. V. Smulders

**Affiliations:** 1 Centre for Behaviour and Evolution, Newcastle University, Newcastle Upon Tyne, NE2 4HH UK; 2 School of Natural and Environmental Sciences, Newcastle University, Newcastle Upon Tyne, NE1 7RU UK; 3 School of Psychology, Newcastle University, Newcastle Upon Tyne, NE2 4HH UK

**Keywords:** Hoarding motivation, Chickadee, Decision rule, Quinine, Palatability, What-where memory, Paridae

## Abstract

Food-hoarding birds hide many different food types, and are able to remember which kind of food they have hidden and where it was located. Usually, these different food types, although potentially of different value to the birds, are all palatable and would be consumed when encountered. We report on the responses of coal tits (*Periparus ater*) to peanut pieces that were made distasteful with quinine. While birds preferred eating normal peanut pieces over quinine-soaked ones, they were still very likely to hoard the distasteful nuts. Birds also did not distinguish between the two nut types when retrieving them after 30 min. These findings point towards the compulsive and automatic nature of hoarding decision, independent of the value of the food being hoarded. We discuss how high hoarding motivation may interact with eating motivation to drive natural patterns of hoarding intensity in the field. Our findings also suggest that the taste of hoarded food items is not part of the representation of the cache memory. We speculate that this may be because tasting the item and caching the item happens in separate locations and are therefore not associated with each other.

## Introduction

Many animals store food for later consumption. Some, like bees and hamsters, gather food and bring it all to a central location that can be defended against competitors. This is called “larder hoarding”. Other species hide each food item (or a small group of items) in a different location, spreading the risk of cache loss. This is called “scatter hoarding” and is typically performed by animals that are unable to defend a central cache. The latter behaviour is typical of a range of bird species, including many in the family Paridae (chickadees and titmice) (Vander Wall 1990).

Much has been written about the ecological function of food hoarding in Parids (e.g., Brodin 2000; Lucas and Walter 1991; Pravosudov and Lucas 2001a, b), as well as about their ability to protect these caches against potential thieves and to retrieve them later (e.g., Baker and Stone 1988; Sherry 1984). However, relatively little has been said about the mechanisms and decision rules that determine whether to hoard an item or not (see Clayton and Dickinson 1999 for work with Corvids). On a large timescale, the patterns are clear: parids show peaks in hoarding behaviour in autumn and sometimes in early spring (Lange et al. 2021; Pravosudov 2006). The mechanisms behind these seasonal changes in behaviour are partially understood. Black-capped chickadees (*Poecile atricapillus*) increase hoarding when days become shorter (Krebs et al. 1995; Shettleworth et al. 1995), independent of gonadal condition (photo-sensitive or photo-stimulated), indicating that this may not relate to sex hormones (MacDougall-Shackleton et al. 2003). One possibility is that hoarding motivation responds directly to the amount of time during which a bird can access food (shorter when days are shorter), but the evidence for this idea remains ambiguous (Karpouzos et al. 2005). What does seem to be clear is that hoarding behaviour increases when energy balance is more precarious.

This is consistent with the evidence for the physiological mechanisms that increase hoarding motivation. Pravosudov (2003) showed that moderate experimental increases in corticosterone, a hormone involved in the stress response and in the physiological access of energy reserves, increase hoarding motivation (as well as eating motivation) in mountain chickadees (*Poecile gambeli*). Baseline corticosterone levels are increased in this species as a consequence of an unpredictable food supply (Pravosudov et al. 2001), but not as a consequence of short days (with ad lib food supply) (Pravosudov et al. 2002). This evidence argues strongly for a direct effect of energetic state on hoarding motivation, in the same direction as it affects feeding motivation. Indeed, recent evidence suggests that direct manipulation of other hormones that are known to affect appetite in birds also affect hoarding motivation in the same direction: injection of ghrelin and leptin decreased both feeding and hoarding in coal tits (*Periparus ater*) (Henderson et al. 2018), although in a separate study, ghrelin did not affect either fat reserves or hoarding (Williamson et al. 2024).

Whereas the general motivation to hoard clearly co-varies with the general motivation to feed on several time scales (from hours to months), this rule must break down on the level of decisions made on individual food items. After all, the act of eating is incompatible with the act of hoarding. So how does a bird decide whether to eat an item or hoard it? In this paper, we describe an unexpected observation from an unrelated experiment, which, together with follow-up studies, gives us insights into hoarding motivation. We show that coal tits (*Periparus* *ater*), despite clearly finding pieces of peanut coated with quinine aversive, will still hoard them. This shows the compulsive, automatic nature of hoarding decisions, as this happens even in the presence of non-aversive food. We also show that the birds are not able to remember which caches were aversive and which were not, giving us insights into the memory for cache contents.

## Study 1

Study 1 was the unsuccessful training phase of a study that never progressed. The original aim was to train the birds that if a feeder was in the same place in space as the feeder on the previous trial, they should open one side of the feeder for a reward, while if the feeder was in a different place in space than the feeder of the previous trial, they should open the other side. Correct choices were rewarded with small peanut pieces, while incorrect choices would result in quinine-soaked peanut pieces, which tits find aversive (Lindstrom et al. 2001).

### Methods

#### Birds and housing conditions

Six adult coal tits were caught in September 2012 using mist nets in Northumberland under Natural England license number 20122488. They were aged 1+ years old based on their plumage (Svensson 1984). A further two coal tits were captured in February 2013. Birds were kept in cloth bird bags for a maximum of two hours and transported to the housing facility. Here, the birds were kept on a 9:15 h light/dark cycle with lights on from 9am to 6pm GMT throughout the course of the study. They were housed individually in cages of 81 cm (W) × 46 cm (D) × 90 cm (H), each of which was equipped with foliage, two perches, a water bowl, a water dispenser, a food bowl and paper bedding. Each cage had two spring-secured, inwardly opening hatches, the lower one for replacing food and water and the upper one to connect the cage to the experiment room during trials. The birds were fed ad libitum on a daily diet of mealworms, sunflower hearts, pine nuts, and peanuts, and bolstered with Orlux© insect pate. During the experimental period peanuts were removed from the home cage food to increase motivation. Immediately prior to all experiments, the birds were food deprived for 1 h 30 min.

All experiments were performed in accordance with ASAB’s guidelines. The research was approved by Newcastle University’s Ethical Review Committee (Project ID no. 309).

#### Experimental environment

The experiment chamber was a white panelled room 216 cm (W) × 350 cm (D) × 235 cm (H) with a door in the centre of each short wall. There was orange plastic netting spread across the upper half of each side wall from which to hang feeders. Further feeders could be hung from two moveable wooden stands with vertical wire mesh attached to them. The room also contained two large (2 m tall) bare forked hardwood tree branches set in concrete blocks. Holes 0.5 cm wide and 1 cm deep were drilled in the trees and feeders, which allowed birds to hoard. Each hole had a wooden 5 cm perch below it. One tree had 8 and the other 14 storing holes. Groups of holes were marked with the same colour tape according to height (e.g. the bottom 5 holes all had green tape, the top 5 holes all had yellow tape) to aid fast identification of the storage site for the observer. For the birds, these just add further landmarks to the shape and position of the trees in the room, but were not sufficient by themselves to identify the holes, as the colour was shared among several holes. Water was available at the base of one of the screens in a bowl. One door connected the experimental environment to the housing room. On the housing room side, this door had a hatch to which the home cages could be attached. This hatch was blocked with a plastic slider that could be inserted or removed, allowing or denying the bird access to the experimental room. The other door contained a large one-way bay window allowing us to observe the birds without disturbing them during trials. This door also provided the experimenters with access to the room to replace feeders etc.

To shuttle the birds between the home cage and the experimental environment, we covered the cage with a thick fabric sheet to darken the cage. We then removed the plastic divider and went to the observation window. At this point, the lights in the experiment chamber were turned on, causing the bird to fly in. When the trial was finished, we extinguished the lights in the experimental room and removed the cover from the cage, allowing the lights from the housing room to shine into the cage and through the hatch. The birds then flew back into the home cage.

#### Experimental procedure

Six coal tits were run in this procedure between 30/11/2012 and 10/12/2012. One coal tit did not collect any nuts and was therefore excluded from the data analysis. The two extra coal tits were run on the same procedure between 4/06/2013 and 17/06/2013. The total sample size was therefore 7 coal tits. Birds experienced between 2 and 4 trials of this type. The data from all trials were combined for each bird. The food items were small pieces of peanut (1–2 mm diameter). Pieces were kept very small so that we could conduct many trials in one session without the birds becoming satiated. To make the food aversive, peanut pieces were soaked in a 2% quinine sulphate solution (similar to Lindstrom et al. 2001); control pieces were soaked in water. Pieces were allowed to dry for 2 days. Quinine tastes bitter, but may also have post-ingestive effects (Skelhorn and Rowe 2007). Whether the effect is purely due to taste or to post-ingestive effects, quinine-soaked nuts are less palatable to the birds. While it is possible that post-ingestive effects may have reduced the motivation to eat generally, there is very little evidence for this in the literature (Skelhorn and Rowe 2007).

**Fig. 1:**
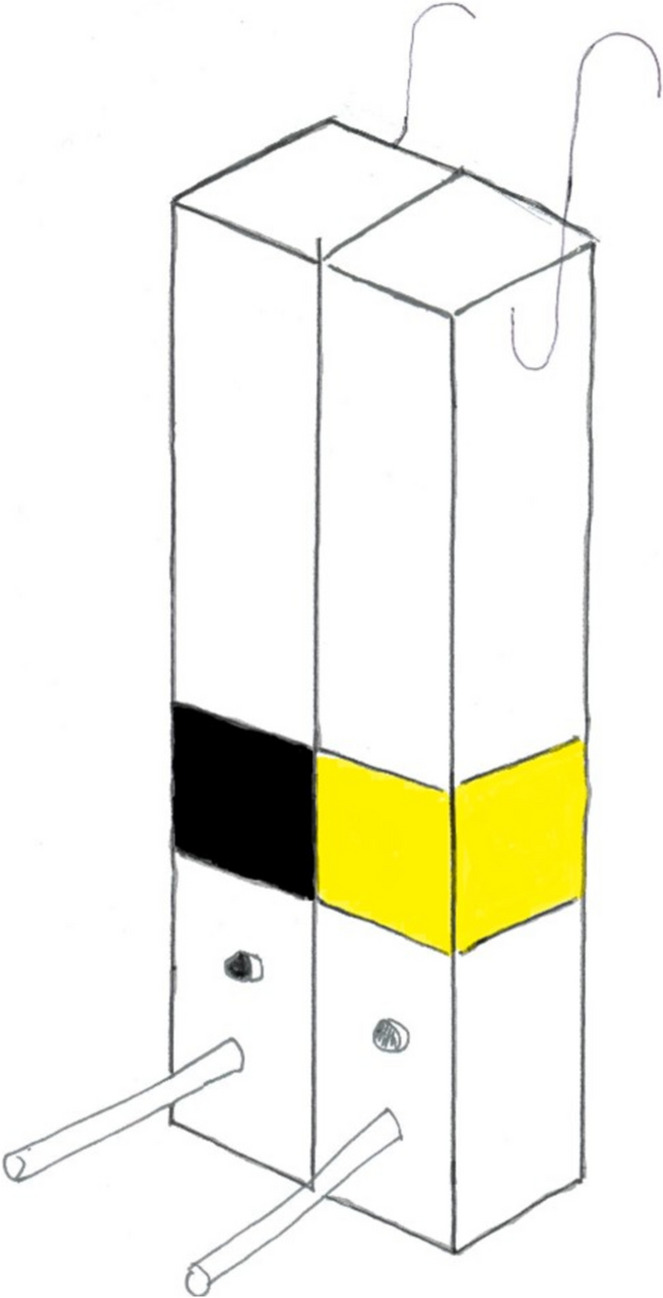
Double feeder with holes for quinine and water soaked peanuts and perches for the birds to land; colour tape marks the different feeders to aid identification for the birds

Food was presented to the birds in feeders made up of two wooden blocks of 25 mm (W) × 25 mm (D) × 200 mm (H), bound together at the top and bottom with elastic bands (Fig. 1). Each wooden block had a 5 mm (D) and 5 mm diameter hole at the bottom with a 5 cm perch underneath it. The left block was marked with yellow electrical tape and the right block with black. In each feeder one quinine-soaked (Q) peanut was placed in one hole and a water-soaked (W) peanut in the other. Which hole contained which nut was dependent on a complex set of rules combining colour and spatial location of the feeder in the room, which proved to be difficult to learn for the birds. One feeder was placed in a random location in the room (randomized from a list of possible locations in a spreadsheet), after which the bird was allowed into the room. After the first feeder was emptied, two new feeders were added to the experiment chamber (one in the same location as before, one in a new location) while the bird was present in the room. Once the bird had removed all pieces of peanut, both feeders were again removed and replaced with two new ones. This process was repeated until the feeders had been replaced 7 times or the bird had been inactive for 30 min.

Each time new feeders were introduced, the time taken for the bird to remove each peanut was recorded. We also recorded the final fate of each peanut piece: fully eaten, dropped on the floor and left there, or hoarded. Note that sometimes, birds would drop a piece of food, but then fly down and continue to manipulate or eat it. In those cases, we only recorded the final fate of the food, not the intermediate events.

#### Data analysis

The fate of peanut pieces was analysed using a Generalized Linear Model approach in SPSS© v.22, using a Poisson distribution with log-transformation for number of seeds collected, and a binomial distribution with log-link function for the proportion of seeds with a given final fate. This procedure produces a Wald’s Chi-square statistic with associated degrees of freedom and *P*-values to test hypotheses. Descriptive statistics are given as mean ± SEM.

### Results

**Fig. 2:**
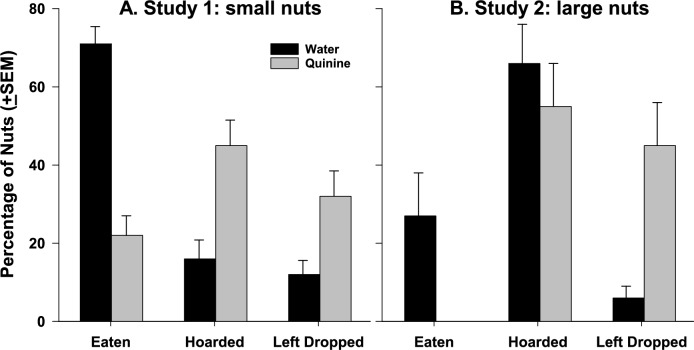
Final destinations of the different nut types in the first two studies. **A** In the first study, we provided small pieces of peanut. A higher proportion of water-soaked (W) nuts were fully eaten than quinine-soaked (Q) nuts, while more Q nuts than W nuts were hoarded, or left on the ground after dropping them. **B** When we provided larger pieces in the second study, still more W nuts were eaten than Q nuts, but this number was much lower than in the first study. Again, Q nuts were more likely to be left on the floor after being dropped than W nuts, but Q and W nuts were equally likely to be hoarded

The birds were slightly, but significantly, more likely to collect water-soaked (W) nuts (25 ± 5) than quinine-soaked (Q) nuts (21 ± 4; *χ*^2^_1_ = 8.575, *P* = 0.003). Once they had taken a nut, W nuts were more likely to be eaten completely (71 ± 4%) than Q nuts (22 ± 5%; *χ*^2^_1_ = 165.79, *P* < 0.0005), while Q nuts were more likely to be left after being dropped (32 ± 6% vs 12 ± 4% of W nuts; *χ*^2^_1_ = 19.13, *P* < 0.0005) and more likely to be hoarded (45 ± 7% vs 16 ± 5% of W nuts; *χ*^2^_1_ = 19.72, *P* < 0.0005; Fig. 2A). However, when looking at the proportion of *uneaten* nuts, there were no differences between the proportion of Q and W nuts that was hoarded (*χ*^2^_1_ = 0.014, *P* = 0.905).

### Discussion

Birds were more likely to finish eating the W nuts than the Q nuts, as would be expected if Q nuts were less palatable than W nuts. They also seemed to have learned something about which taste to expect in which location, since they were more likely to collect W nuts than Q nuts from the feeders. Although they clearly preferred to eat W nuts over Q nuts, they hoarded both with equal likelihood if they were not fully eaten. This was surprising, because once they noticed the nuts were aversive, the birds could have just dropped the remaining Q nuts and left them instead of hoarding them. This shows that, despite preferring not to eat aversive food items, coal tits will still store them.

## Study 2

Study 2 was designed as a follow-up experiment to replicate our findings that coal tits will hoard Q nuts equally likely as W nuts, and to test whether they remember which type of nut they hid in which location. We also wanted a more sensitive measure of palatability, so we gave them larger pieces of peanut in this study to measure the time spent nibbling on the two nut types.

### Methods

#### Birds and housing conditions

The same birds were used as in Study 1, with the exception of one bird, which died unexpectedly. One other coal tit again did not hoard any nuts and was therefore excluded from the analysis. Four birds were run between 12/12/2012 and 19/12/2012, and two birds were run under the same conditions between 14/06/2013 and 24/06/2013, for a total sample size of 6 coal tits. Housing conditions were identical to Study 1.

#### Experimental environment

The same room was used as in Study 1. During this study, the room was set up with 5 trees with a total of 52 potential cache sites. A pre-determined coordinate system allowed us to track the location of each hoarded seed. The position of the trees was changed from trial to trial to prevent interference among trials.

#### Experimental procedure

Ten 5-mm chunks of each peanut type (W and Q) were presented on a wooden platform near the observation window. The two nut types were separated by a low barrier to prevent them from mixing together so that the observers could tell which nut type was collected by the birds. The side of the platform used for the Q nuts was alternated between sessions. The birds were allowed to feed ad libitum from the platform until all peanuts had been taken, until the birds were inactive for 10 min, or until a maximum of 1 h had elapsed. Birds that had hoarded nuts were kept in the home cage (without food) for 30 min after the hoarding session, during which time the room was swept and all food items removed, except for the hoarded nuts in their cache sites (including sites that were not the pre-drilled holes). The bird was then allowed to re-enter the chamber and retrieve any previously hoarded foods until all food had been retrieved, until the bird was inactive for 10 min, or until a maximum of 30 min had elapsed. We ran three such hoarding and retrieval trials for each bird.

We observed which nut types were collected by each bird, as well as the final fate of each nut (as in Experiment 1). During retrieval, we also noted which nut types they retrieved. For all nuts, we recorded the cumulative time the birds spent nibbling at each nut piece. The timer was stopped when the bird moved around with the nut piece in its beak and restarted when the nut was put back under the bird’s feet and the bird started nibbling at it.

#### Data analysis

Analysis was similar to Study 1, with the addition of an analysis of nibbling times. Nibbling times were analysed using Linear Mixed Models with time as a continuous dependent variable without transformation, bird as a random factor and nut type and nut fate as a within-subject factors.

### Results

In Study 2, surprisingly, the birds were more likely to collect Q nuts (23 ± 5) than W nuts (19 ± 4; *χ*^2^_1_ = 9.014, *P* = 0.003). Once a nut was picked up, it was very unlikely to be fully eaten, whether it was a W or a Q nut. The number of W nuts to be completely consumed ranged from 1 out of 29 (3%) to 4 out of 6 (67%) and no Q nuts were ever fully consumed. As in Study 1, fewer W nuts were left on the floor (8 ± 3%) than Q nuts (36 ± 8%; *χ*^2^_1_ = 38.17, *P* < 0.0005). Finally, in this study, the birds hoarded a significantly higher proportion of W nuts (66 ± 9%) than Q nuts (55 ± 10%; *χ*^2^_1_ = 8.54, *P* = 0.003; Fig. 2B), and this difference is even higher when just considering uneaten nuts (Q: 64 ± 8%; W: 89 ± 4%; *χ*^2^_1_ = 50.44, *P* < 0.0005).

**Fig. 3:**
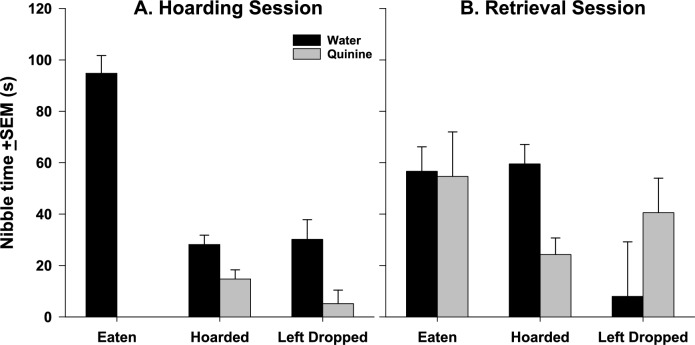
Nibbling times on different nut types during the hoarding phase (**A**) or the retrieval phase (**B**) of Study 2. Initially, birds spend longer nibbling on W nuts than Q nuts, and longer on nuts that are finished than on nuts that are hoarded or dropped. At retrieval, birds spend equal amounts of time nibbling on both nut types before they are fully eaten, but more time on W nuts than Q nuts before rehoarding them

We also recorded how long birds spent nibbling at nuts before fully ingesting, hoarding or dropping them. Coal tits nibbled for a significantly shorter amount of time on Q nuts than on W nuts (*F*_1,130_ = 13.54, *P* < 0.001), and they nibbled longer on nuts they fully ingested than either those they hoarded or dropped (*F*_2,130_ = 34.19, *P* < 0.001; Fig. 3A). The difference in nibble time between hoarded and dropped nuts was not significant (*P* = 0.463).

During the retrieval phase, the coal tits were equally likely to retrieve Q nuts as W nuts (*χ*^2^_1_ = 0.083, *P* = 0.773), retrieving about 1/3 of each across all sessions. Of those retrieved, W nuts were equally likely to be completely eaten as Q nuts (*χ*^2^_1_ = 2.88, *P* = 0.090). There was also no significant difference between Q and W nuts in how likely they were to be dropped and left (*χ*^2^_1_ = 0.54, *P* = 0.463) or rehoarded (*χ*^2^_1_ = 1.49, *P* = 0.222). Across both nut types, 66% of retrieved nuts were rehoarded. How much time birds spent nibbling on a retrieved nut depended on both the nut type and the eventual fate of the nut (main effects not significant; interaction: *F*_2,52_ = 3.78, *P* = 0.029). The time spent nibbling on nuts that were completely eaten was the same for the two nut types. However, the birds spent less time nibbling on Q nuts that were eventually rehoarded than on W nuts that were eventually rehoarded. Very few nuts were dropped, but for those, birds spent very little time nibbling on W nuts that were then abandoned, while they spent more time on Q nuts that were then abandoned (Fig. 3B).

### Discussion

Our second study showed again that coal tits are very likely to hoard quinine-soaked peanut pieces, although when the pieces are larger and less likely to be fully eaten, they were more likely to hoard W nuts. In the hoarding sessions, birds did spend longer nibbling on W nuts than Q nuts before hoarding them, again indicating that Q nuts are less palatable. Birds were also much more likely to abandon dropped Q nuts than dropped W nuts.

We saw no evidence that the birds remembered the nut type hoarded, as both were equally likely to be retrieved. At retrieval (with no other nuts in sight), some of the Q nuts were fully consumed, and they took the same amount of time to be finished as W nuts. This indicates that birds are more likely to accept poor-tasting food if there are no obvious alternatives available. If they really do not remember which nuts are bad tasting and which good tasting, then it also would not make sense to drop the nut and retrieve another one, because the next nut may taste just as bad. Still, Q nuts were more likely to be abandoned, and less time was spent eating them before rehoarding them than W nuts. This indicates that after 30 min, the Q nuts remained less palatable than the W nuts.

We were surprised to find that the birds did not show memory for the content of the caches, as black-capped chickadees have been shown to remember the content of their caches (Feeney et al. 2009, 2011; Sherry 1984). We find it unlikely that the birds remembered which item was hidden and where, yet did not take this information into account when retrieving the nuts, as they clearly still treated the Q nuts as less palatable than the W nuts. Therefore, we hypothesize that, unlike for visual cues of food types, the birds are not able to associate taste cues with the cache locations. We discuss why this might be in the general discussion.

## Study 3

Study 3 aimed to test whether birds could remember the locations of Q nut caches if the Q nuts were visibly distinguishable from W nuts.

### Methods

#### Birds and housing conditions

The same birds were used as in the previous experiments, and their housing conditions were identical. All birds started training to associate a colour with quinine, but only 3 birds reached criterion within the allotted time (see below), so only 3 birds were used in the actual experiment.

#### Experimental environment

The environment was identical to Experiment 2 (with trees moved around again to reduce interference).

#### Experimental procedure

Before the start of the experiment, the birds were first trained to associate a colour with quinine. Peanut pieces were dyed either green or blue by soaking for 24 h in a 50:50 mixture of food colouring and water, then allowed to dry before undergoing the same soaking process as in the previous experiments in either 2% quinine sulphate solution or water. Three birds were assigned blue as their control and green as the bitter tasting food, while the other three were assigned the opposite. Birds were then trained in their home cages to learn the association between colour and taste. We added 5 nuts of each type to their daily food bowl for three days and allowed them to feed ad libitum. After this, birds were food restricted as before and given two nuts of each type. We continued training and daily testing until the birds correctly chose the water soaked peanut first, for three consecutive days. Three birds learned this rule (our records do not specify which colour they were trained on) within 6 days and they were tested in three trials, identical to Experiment 2, but each lasting a maximum of 30 min, and with coloured, instead of uncoloured peanut pieces.

#### Data analysis

Data analysis was as in Study 2.

### Results

Although all birds collected a sufficient number of W nuts (from 6 to 21 out of 30 offered over three trials), not a single Q nut was selected by any of the birds (*χ*^2^_1_ = 198.48, *P* < 0.0005). No retrieval data were analysed since the birds never took or hoarded any Q nuts.

### Discussion

Although the study was designed to test the hypothesis that the bird should be able to remember which cache contained W nuts and which Q nuts if they were visually distinct, the fact that none of the birds ever picked up a single Q nut made this impossible. The results do tell us, however, that when birds picked up Q nuts before, and nibbled at them, they most likely did this without knowing ahead of time they would be bitter tasting. Nibbling then allowed them to discover this.

## General discussion

As we expected, all the data suggest that quinine is aversive to coal tits and that the birds prefer not to eat bitter nuts if they have the option. It was therefore surprising that they still hoarded a large number of quinine-soaked nuts, and even more surprising that they could not remember which of the caches contained quinine-soaked nuts. Here we discuss why the birds would hoard unpalatable food, and why they might not be able to remember which caches were unpalatable.

### Implications for hoarding motivation

The comparative cognition literature on hoarding decisions can give the impression that the decision to hoard an item is usually “well-considered” and rational in the moment (Clayton and Dickinson 1999; Correia et al. 2007; Grodzinski and Clayton 2010; but see also van der Vaart et al. 2012). However, those who work with food-hoarding birds know how easy it is to induce the behaviour, even in captive conditions. This points to the highly compulsive and automatic nature of the behaviour. Our findings add to this picture: even when birds have collected a food item that is unpalatable, but nevertheless edible, the next decisions is not to drop it and continue foraging for better nuts (and there were plenty of nuts still on the table), but to hoard the nut they had collected. Only if the nut was dropped were they more likely to find a new nut if the nut was of low palatability. We saw the same thing again after retrieval of unpalatable nuts: if the nut was not completely eaten, it was rehoarded. The motivation to hoard a food item once it is in the possession of the bird applies equally to newly collected items and to retrieved caches. While this is a mechanistic explanation, it is easy to see the selective benefit of such a decision rule, as unpalatable food may be better than no food at a later retrieval time.

If hoarding motivation is so high and so compulsive, how then do the birds make the decision about whether to hoard a food item or to eat it? We hypothesize that this may be a case of conflicting motivations, and that whichever motivation is highest wins out. As such, we see that birds will often start eating a nut but then finish by hoarding it. This implies that the initial eating may quickly reduce the eating motivation, at which point the always-high hoarding motivation wins out and the bird hoards what is left of the nut. Such a system of high constantly high hoarding motivation may also be behind seasonal patterns in hoarding intensity (Pravosudov 2006). The start of longer, colder nights in the autumn is likely to increase the motivation to forage (e.g. because of higher use of internal fat reserves; Keen-Rhinehart et al. 2010). When food is abundant (in autumn), hoarding motivation will outcompete eating motivation most of the time (as there is always food to be found), leading to high hoarding intensity. Foraging and hoarding motivation remain high in the winter, but at this time of year, food availability is lower, so for any food found, the motivation to eat is usually higher than the motivation to hoard (because birds will be less likely to be satiated). Therefore, hoarding intensity is reduced dramatically, despite high hoarding motivation. This hypothesis about the mechanism behind seasonal patterns in hoarding intensity remains to be tested, e.g. by providing ad lib food in late winter, when days are similar in length to early autumn, but natural food availability is much lower. We would predict that birds should again hoard more intensely if given enough food to satisfy their eating motivation. Informally, this is evident at each bird feeder in winter and during mast years in the field (Pravosudov 2006).

### Implications for cache memory

Although quinine-soaked nuts were clearly less palatable than water-soaked nuts, the birds did not differentiate between the two types when retrieving their caches. This implies that they did not remember which nut type had been cached in which locations. This is surprising, as previous studies have shown that Parids can remember which food type was hidden in which location, even when both are palatable (Feeney et al. 2009, 2011; Sherry 1984). One big difference is that in previous studies, the birds always hoarded two visually distinct kinds of food (sunflower vs. safflower seeds; or sunflower seed vs. mealworms), while in our study, all the food was peanut pieces. We speculate that the difference may be in the type of information that is being remembered. We can see two possible explanations.

One possibility is that taste is more difficult to associate with a cache location than visual information. However, there is evidence that rats can associate taste with spatial locations. For example, rats can be trained to go to a specific location in response to a specific taste (Gautam et al. 2012). This is of course not the same as associating a taste with the location in which it was experienced, but rats can do that as well. Rat hippocampal place cells encode information not only about the location where the rat finds itself, but also about the value of a taste experienced in that location (Herzog et al. 2020, 2019). In addition, rats can be associate taste with locations on a circular track, to such a degree that the representation of space is influenced by the location where the taste is experienced (Nair and Roy 2021). Place cells have also been recorded in food-hoarding birds (Chettih et al. 2024; Payne et al. 2021), although it is unknown whether these can also encode information about taste.

A second possibility is that taste is not associated with the cache locations because the taste was not experienced at the cache location itself. Typically, the bird will be eating the nut in one location and then fly somewhere else to hide it. The distribution of taste buds in many birds does not include the front of the tongue or the hard parts of the beak (Niknafs et al. 2023). It is therefore very likely that coal tits only taste the food while they are nibbling bits off it and swallowing them. When the seed is held in the beak for manipulation during hoarding and flying around, they are likely not to taste the seed at all, and the information is therefore not present to be encoded with the spatial information. When birds remember different food types as more or less palatable, it is most likely that they have already made an association between the taste and the appearance of those foods in the past, so that just the memory of the appearance is enough to drive the published results on memory for cache content in Parids (Feeney et al. 2009, 2011; Sherry 1984), while in our study, the appearance of the two nut types did not differ.

## Conclusion

Coal tits, like many hoarding birds, have a strong motivation to hoard food, even if the food they have collected is less palatable. This high hoarding motivation is sometimes outcompeted by other motivations, and the interplay among different motivations may explain the patterns of hoarding intensity in the field. Despite being able to distinguish palatable from less palatable food once it has been collected, the birds did not remember which type of food was hoarded in a particular location. We hypothesize that this is because the taste is not experienced at the cache site itself.

## Data Availability

The data can be accessed at 10.25405/data.ncl.27249690.v1.
