# Correction: Responses of coal tits (*Periparus ater*) to aversive food: insights into hoarding motivation and memory

**DOI:** 10.1007/s10071-025-01986-7

**Published:** 2025-07-26

**Authors:** D. D. O’Hagan, D. Donley, S. W. Y. Yeung, C. D. Blasi Foglietti, D. Wales, D. Wintersgill, T. V. Smulders

**Affiliations:** 1 Centre for Behaviour and Evolution, Newcastle University, Newcastle Upon Tyne, NE2 4HH UK; 2 School of Natural and Environmental Sciences, Newcastle University, Newcastle Upon Tyne, NE1 7RU UK; 3 School of Psychology, Newcastle University, Newcastle Upon Tyne, NE2 4HH UK

**Correction: Animal Cognition (2025) 28:44**

10.1007/s10071-025-01969-8

In this article the ‘Conflict of Interest’ statement was missing and should have been “Editor T. V. Smulders is a guest editor for the journal and has not taken part in the manuscript’s peer review process”.

The original article has been corrected.

